# Normal Cerebral Oxygen Consumption Despite Elevated Cerebral Blood Flow in Adolescents With Bipolar Disorder: Putative Neuroimaging Evidence of Anomalous Energy Metabolism

**DOI:** 10.3389/fpsyt.2019.00739

**Published:** 2019-10-11

**Authors:** Sudhir Karthikeyan, Lisa Fiksenbaum, Anahit Grigorian, Hanzhang Lu, Bradley J. MacIntosh, Benjamin I. Goldstein

**Affiliations:** ^1^Centre for Youth Bipolar Disorder, Sunnybrook Health Sciences Centre, Toronto, ON, Canada; ^2^Department of Radiology, Johns Hopkins University School of Medicine, Baltimore, MD, United States; ^3^Department of Medical Biophysics, University of Toronto, Toronto, ON, Canada; ^4^Physical Sciences, Sunnybrook Research Institute, Toronto, ON, Canada; ^5^Heart and Stroke Foundation Canadian Partnership for Stroke Recovery, Sunnybrook Health Sciences Centre, Toronto, ON, Canada; ^6^Department of Pharmacology, University of Toronto, Toronto, ON, Canada; ^7^Department of Psychiatry, University of Toronto, Toronto, ON, Canada

**Keywords:** bipolar disorder, adolescent, cerebral metabolic rate of oxygen, CMRO_2_, cerebral blood flow, venous oxygenation, TRUST MRI

## Abstract

**Background:** Regional cerebral blood flow (CBF) is reportedly altered in both adolescents and adults with bipolar disorder (BD). Whether these CBF differences are part of an overall imbalance in cerebral energy homeostasis remains unknown. Therefore, we examined global cerebral metabolic rate of oxygen consumption (CMRO_2_) as a physiological index of brain metabolism in adolescents with and without BD.

**Methods:** One hundred and fifteen adolescents (mean age 17.3 ± 1.4 years), including 58 BD (type I, II, or not otherwise specified [NOS]) and 57 age-matched healthy controls (HCs) participated in this magnetic resonance imaging (MRI) study. Global estimates for venous blood oxygenation (Y_v_) and grey matter CBF were measured using T2-relaxation-under-spin-tagging (TRUST) and arterial spin labeling (ASL) MRI, respectively. CMRO_2_ was calculated using the Fick principle of arteriovenous difference to test for a group difference. We also examined CMRO_2_ in relation to mood states (i.e. euthymic, depressed, or hypomanic/mixed).

**Results:** Although CBF was significantly higher in BD compared to HCs, there was no group difference in global CMRO_2_, nor Y_v_. Meanwhile, Y_v_ significantly decreased with age, and females tended to have greater CBF and CMRO_2_ in comparison to males. Lastly, there was no significant association between CMRO_2_ and mood states.

**Conclusions:** Our results indicate a potential mismatch between cerebral blood supply and oxygen metabolism in BD, suggesting inefficiency in energy homeostasis in the brain. Mapping CMRO_2_ would provide the spatial resolution to investigate regional alterations in metabolism, particularly in the brain regions where CBF is increased.

## Introduction

Bipolar disorder (BD) is a severe chronic mood disorder associated with an increased risk of developing premature cardiovascular disease (CVD) (1, 2). Indeed, CVD is the leading cause of mortality in BD, despite the increased risk of suicide and prevalence of other comorbidities (3, 4). In recent years, there has been increasing support for the notion regarding vascular pathology as an important neurobiological underpinning of BD (1, 5). This link between vascular pathology and BD is further evidenced by research showing alterations in cerebral blood flow (CBF) in individuals with BD (6). CBF is an important physiological parameter which reflects the supply of oxygen and glucose to the brain and is known to be associated with cardiometabolic risk factors (7). Although the human brain accounts for just 2% of total body mass, it consumes about 20% of the total energy (8). Thus, changes in blood supply may affect energy homeostasis in the brain that is crucial for normal neuronal functioning.

Most studies that have examined CBF in adults with BD have found decreased CBF in frontal and temporal brain regions in BD participants, particularly during depressive episodes, compared to healthy controls (HCs) (6, 9–11). In contrast, the sole study that has investigated CBF in adolescents with BD found region-specific elevation in CBF (12). These findings raise questions as to how alterations in cerebral hemodynamics affect energy homeostasis in the brain. Further research is needed to clarify the relationship between CBF changes and energy homeostasis in individuals with BD.

Multiple lines of evidence suggest that energy metabolism is disturbed in BD and is associated with mitochondrial dysfunction. Early studies using magnetic resonance spectroscopy (MRS) reported a reduction in phosphocreatine levels in the brains of BD patients, indicating inefficient adenosine triphosphate (ATP) synthesis (13, 14). Subsequent genetic studies found that BD is associated with mitochondrial DNA (mtDNA) mutations and polymorphisms (15, 16). In addition, post-mortem brain studies have shown abnormal mitochondrial morphology (17), reduction in mitochondrial protein function, and increased oxidative damage (18) in brains of patients with BD. Furthermore, studies have detected increased lactate concentration in the brain (19) and cerebrospinal fluid of individuals with BD (20), suggesting a shift from oxidative phosphorylation to glycolysis. Consistent with this, findings from positron emission tomography (PET) studies showing altered cerebral glucose metabolism in BD (17, 18) provide additional support for the view that abnormal energy metabolism is a key factor in the pathophysiology of BD.

Cerebral metabolic rate of oxygen consumption (CMRO_2_) is a measurable index of oxygen utilization in the brain. It is defined as the amount of oxygen consumed per unit mass tissue and per unit time, and reflects oxygen demand in the brain. Regulation of oxygen metabolism is vital for normal neuronal functioning. Altered CMRO_2_ is reported in studies on aging (21, 22) as well as in several disease states including Alzheimer’s (23), Parkinson’s (24), and multiple sclerosis (25).

CMRO_2_ is related to the difference in oxygen saturation from artery to vein, known as the oxygen extraction fraction (26). Oxygenated blood that passes through the veins is the venous oxygenation (Y_v_). Until recently, most studies have relied on PET combined with blood sampling to measure Y_v_ and calculate CMRO_2_. The use of a radioactive tracer and the invasive nature likely limited its feasibility, especially in children and adolescents. This is reflected by a lack of studies examining brain physiology in youth.

The aim of the present study was to compare CBF, Y_V_, and CMRO_2_ in adolescents with and without BD. We used a validated T2-relaxation-under-spin-tagging (TRUST) magnetic resonance imaging (MRI) technique to noninvasively measure Y_v_ (27, 28). We also evaluated the relationship between CBF, Y_V_, and CMRO_2_ and mood states (i.e. euthymic, depressed, or hypomanic/mixed). We hypothesized that BD and HC participants will show differences in both CBF and the rate of oxygen utilization.

## Methods

### Participants

One hundred and fifteen English-speaking adolescents between the ages of 13 and 20 years of both sexes and any race/ethnicity were included (58 BD, 57 HCs). BD participants who met criteria for BD [I, II, or not otherwise specified (NOS)] were recruited from a tertiary sub-specialty clinic at Sunnybrook Health Sciences Centre in Toronto, Ontario. HCs without any major mood or psychiatric disorders (i.e. no lifetime mood or psychotic disorders, no recent anxiety disorder or alcohol/drug dependence in the past 3 months) and no first- or second-degree family history of BD or psychotic disorder were recruited from the community. Participants were excluded if they met any of the following criteria: i) unable to provide informed consent; ii) existing cardiac condition, auto-immune illness, or inflammatory illness; iii) currently taking anti-inflammatory, anti-lipidemic, anti-hypertensive agents; iv) contraindications to MRI (e.g. cardiac pacemaker or other implanted device); v) neurological or cognitive impairment; and vi) infectious illness within the past 14 days. Written informed consent was obtained from both participants and their parent/guardian prior to any procedures. All procedures were approved by the research ethics board at Sunnybrook Health Sciences Centre. A semi-structured diagnostic interview was used to assess study requirements.

### Clinical Procedures and Measures

Psychiatric diagnosis was determined using the Schedule for Affective Disorders and Schizophrenia for School Age Children, Present and Life Version (K-SADS-PL) (29). The K-SADS Depression Rating Scale (DEP-P) (30) and K-SADS Mania Rating Scale (MRS) (31) were used in place of the mood sections of the K-SADS-PL to assess current and lifetime symptoms of depression and mania. Symptom scores during the worst week in the past month were used as a measure of current mood state. Information provided by the participant, as well as their parent/guardian, was incorporated into the final summary score for each scale. BD-I and BD-II diagnosis was determined using the *Diagnostic and Statistical Manual of Mental Disorders, 4* *^th^* *Edition* (*DSM-IV*), criteria (32). BD-NOS was operationally defined using criteria outlined in the Course and Outcome of Bipolar Youth (COBY) study (33): i) two *DSM-IV* manic symptoms (three if only irritable mood is reported), ii) change in functioning, iii) mood and symptom duration of at least 4 h during a 24 h period, and iv) at least four cumulative 24 h periods of episodes that meet the mood, symptom, and functional change criteria over the participant’s lifetime.

### Overview of MRI Procedures

MRI was performed on a 3 T MR scanner (Philips Achieva, Philips Healthcare, Best, NL) using an eight-channel phased array RF head coil. The imaging protocol included T1-weighted images for anatomical registration, pseudo-continuous arterial spin labeling (PC-ASL) for CBF measurement, and TRUST for Y_v_ quantification.

#### Anatomical Imaging

Anatomical T1-weighted imaging was performed with high-resolution fast-field echo imaging [repetition time (TR)/echo time (TE)/inversion time (TI)] = 9.5/2.3/1,400 ms, field of view 240 × 191 mm, spatial resolution 0.94 × 1.17 × 1.2 mm, 256 × 164 × 140 matrix, scan duration 8:56 min:s). T1-weighted images were skull-stripped, co-registered to ASL space and standard space, normalized, and parcellated based on tissue type. All of the steps above were performed using Functional Magnetic Resonance Imaging of the Brain (FMRIB) Software Library (FSL) tools.

#### Global CBF

Gray matter (GM) CBF for the whole brain was measured using ASL. Phase contrast angiography scout images were acquired to visualize the position of the carotid and vertebral arteries that corresponded to the ASL labeling plane, and thereby facilitate ASL quality control. ASL data were acquired with the following parameters: single-shot two-dimensional echo-planar imaging (EPI) (TR/TE = 4,000/9.7 ms, 64 × 64 × 18 matrix, spatial resolution 3 × 3 × 5 mm), 1,650 ms labeling duration, post-label delay of 1,600 ms for the most inferior slice, 30 control-tag image pairs of unlabeled and labeled arterial blood water, and scan duration of 4:08 min:s.

Processing of ASL data was done using FSL tools (12). Images were first co-registered to a reference volume. Signal differences between consecutive control and tag images were obtained to measure the amount of perfused labeled arterial blood and thus provide relative CBF estimates. Signal in GM was optimized by removing images with excess head motion. Each individual’s difference images were converted into a quantitative CBF map by relying on physiological and MR parameters, such as relaxation rates and transit times of the labeled arterial blood, to provide absolute units for the image intensity (ml/100 g/min) (34). A 5 mm smoothing kernel was applied to the CBF maps. GM global signal was reported based on mean CBF values extracted from GM masks that were segmented from the T1-weighted images and subsequently registered to ASL space.

#### Global Venous Oxygenation

Global cerebral venous oxygenation saturation, Y_v_, was measured in the superior sagittal sinus (SSS) using the validated TRUST MRI technique (28, 35). First, a single oblique axial imaging slice was positioned parallel to the anterior-commissure posterior-commissure line 20 mm above the sinus confluence. TRUST MRI was performed with the following parameters: voxel size 3.44 × 3.44 × 5 mm^3^, TR = 3,000 ms, TI = 1,022 ms, labeling thickness = 100 mm, gap = 22.5 mm, effective TE = 0, 40, 80, and 160 ms, and scan duration of 1:20 min:s.

TRUST data were processed using MATLAB scripts as described previously by Lu and colleagues (28, 35). First, a venous blood signal was extracted by pairwise subtraction of control and labeled images. A preliminary region-of-interest (ROI) mask was then manually drawn on the difference images to include the SSS. Within the ROI, four voxels showing the largest difference signals were selected for spatial averaging. A monoexponential function was used to fit the average venous blood signal as a function of TE and thereby obtain a T2 estimate, which was then converted to Y_v_ using a calibration curve. Hematocrit values for T2 calibration were based on laboratory reference values (LifeLabs, Ontario, Canada) accounting for both age and sex (36).

### Global CMRO_2_

Global CMRO_2_ was calculated using the Fick principle in units of μmol O_2_/100 g/min (26):

$$CMRO_{2}=CBF*(Y_{a}-Y_{v})*C_{a}$$

CBF values were extracted as the average CBF level within the grey mask on a per-participant basis. This single ROI served as a global CBF estimate. Y_v_ was also calculated on a per-participant basis, from within the SSS that drains a large proportion of the venous blood in the brain. Arterial oxygenation (Y_a_) saturation, which is close to unity, was estimated to be 99% for all participants as it is minimally affected by age (21, 37). The value for C_a_, which represents the amount of oxygen molecules that a unit volume of blood can carry, was based on physiological literature and assumed to be 833.7 μmol O_2_/100 ml blood (38).

### Statistical Analysis

All analyses were performed using IBM SPSS Statistics for Windows, version 25 (IBM Corp., Armonk, NY, USA). Comparison of demographic and clinical characteristics between groups was assessed using t-tests or chi-square tests, as appropriate. Group differences in global measures of CBF, Y_v_, and CMRO_2_ were investigated using analysis of covariance (ANCOVA), including age and sex as covariates, as these variables are known to have an effect on the physiological parameters (21, 22, 28). Pearson’s correlation was used to investigate the associations between continuous variables. Significance was set at *p* < 0.05 for all analyses, and values are reported as means ± standard deviation (SD).

## Results

### Demographic and Clinical Characteristics

**Table 1** presents demographic and clinical characteristics. The majority of participants were female (57%) and Caucasian (70%) with a mean age of 17.3 ± 1.40. There were no significant age, race, or sex differences between groups. Mean body mass index (BMI) was significantly higher in BD (24.04 ± 4.51 kg/m^2^) than HCs (21.44 ± 2.79 kg/m^2^; *t* = 3.73, *p* = 0.012, *d* = 0.69). As expected, there were multiple significant differences in clinical characteristics such as mood symptoms, medication, and lifetime history of cigarette smoking.

**Table 1:** Demographic and clinical characteristics.^a^

Characteristics	Participants		Statistics	
	BD (N = 58)	HCs (N = 57)	T/χ^2^	P-value
**Sociodemographics**				
Age, years	17.48 ± 1.25	17.05 ± 1.51	1.65	0.10
Age of BD onset, years	14.98 ± 2.33	—	—	—
Caucasian, n (%)	44(75.9)	36(63.2)	2.19	0.14
Sex (females), n (%)	32(55.2)	33(57.9)	0.09	0.77
SES, mean rank	56.80	59.22	1,583.50^#^	0.66
**BD subtype, n (%)**				
BD-I	18(31.0)	—	—	—
BD-II	19(32.8)	—	—	—
BD-NOS	21(36.2)	—	—	—
**Clinical characteristics**				
Smoking, n (%)	7(12.1)	0	7.33	0.007*
BMI	24.04 ± 4.51	21.44 ± 2.79	3.73	0.001*
History of depressive episodes, n (%)	47(81.0)	—	—	—
**Current medication, n (%)**				
SGA	35(60.3)	—	—	—
Lithium	13(22.4)	—	—	—
Antidepressant (SSRI)	6(10.3)	1(1.8)	3.71	0.054
Antidepressant (non-SSRI)	2(3.4)	0(0)	2.00	0.16
Stimulants	5(8.6)	3(5.3)	0.50	0.48
**Clinical scores (±)**				
Mania score—current	8.97 ± 10.24	0.12 ± .60	20.57	<0.001*
Mania score—lifetime	30.55 ± 10.98	0.63 ± 1.47	6.57	<0.001*
Depression score—current	15.45 ± 11.77	0.68 ± 1.81	17.45	<0.001*
Depression score—lifetime	30.62 ± 12.45	1.53 ± 2.64	9.44	<0.001*

a Values are mean ± SD or frequency. BD, bipolar disorder; BD-I, bipolar I disorder; BD-II, bipolar II disorder; BD-NOS, bipolar disorder not otherwise specified; HCs, healthy controls; SES, Socioeconomic status; BMI, body mass index; SGA, second-generation antipsychotic; SSRI, selective serotonin reuptake inhibitor. ^#^, Mann–Whitney U statistic, * = significant difference.

### Global CBF

**Figure 1:**
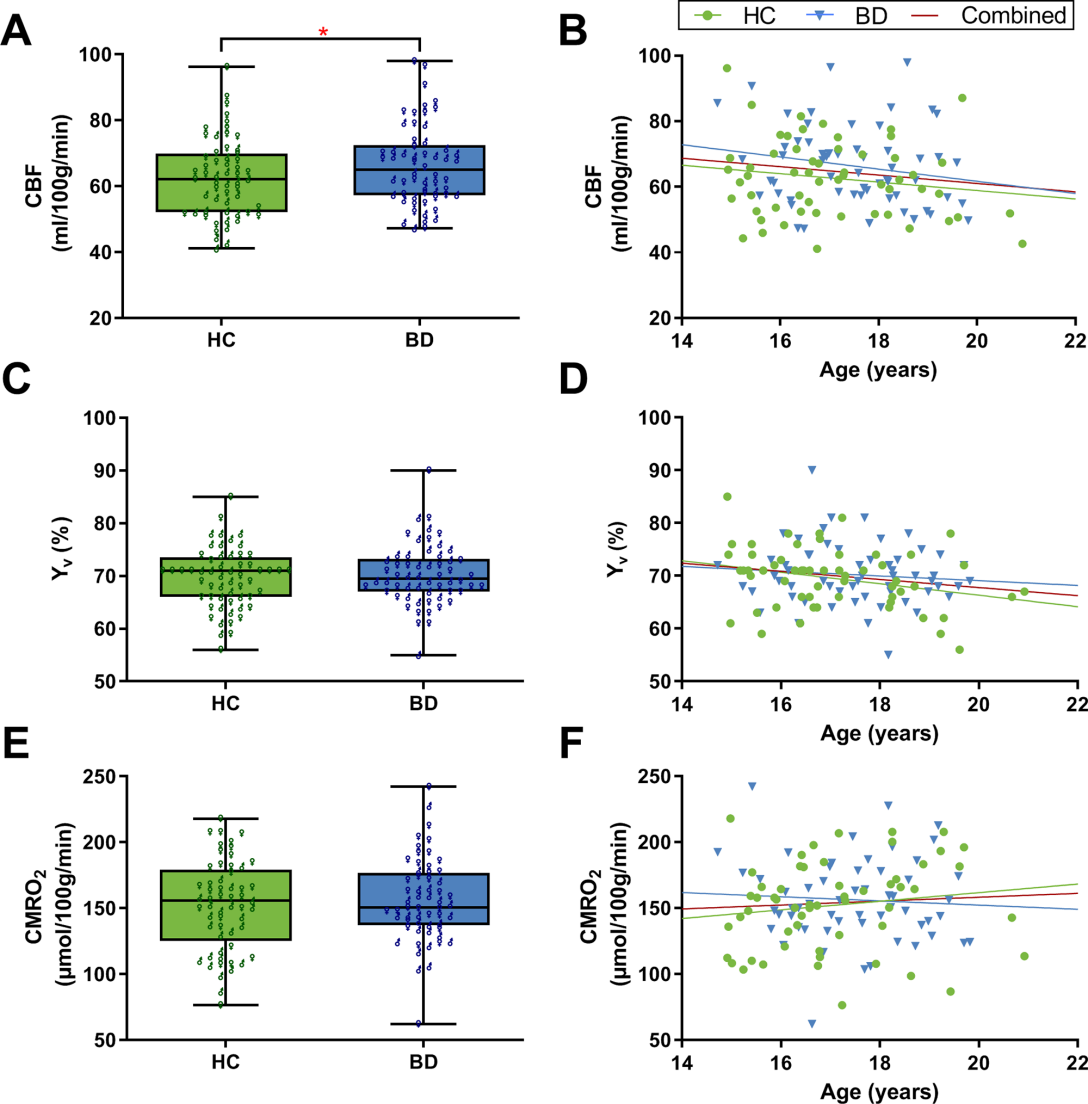
Global grey matter cerebral blood flow (CBF), venous oxygenation (Y_v_), and cerebral metabolic rate of oxygen (CMRO_2_). **(A)** The bipolar disorder (BD) group had significantly greater CBF compared to healthy controls (HCs) (*F* = 4.51, *p* = 0.036). CBF was significantly greater in females compared to males (*t* = 3.76, *p* < 0.001). **(B)** There was no significant association between age and CBF. **(C)** Y_v_ did not significantly differ between BD and HCs. **(D)** Correlation analysis showed that age has a significant effect on Y_v_; *r* = −0.19, *p* = 0.045. **(E)** CMRO_2_ did not significantly differ between BD and HCs. Female participants overall had greater CMRO_2_ compared to males (*t* = 4.51, *p* < 0.001). **(F)** There was no significant association between age and CMRO_2_. Panels A, C, and E are box plots with superimposed data points showing distribution of values in BD (n = 58) and HCs (n = 57). Box = 25^th^ and 75^th^ percentiles; horizontal line = median, bars = min and max values; ♂ = male, ♀ = female. Panels B, D, and F are scatter plots showing the relationship between the physiological parameters and age (N = 115). Each marker represents data from one subject.

There was a significant effect of sex (*t* = 3.76, *p* < 0.001, *d* = 0.69). Overall, females had greater CBF in comparison to males (67.93 ± 12.82 vs. 60.08 ± 9.56 ml/100 g/min, respectively). The BD group had significantly greater global CBF in comparison to HCs, when controlling for age and sex (66.35 ± 12.18 vs. 62.64 ± 11.87 ml/100 g/min, respectively; *F* = 4.51, *p* = 0.036, η*_p_^2^* = 0.039; **Figure 1A**). Sex was a significant covariate (*F* = 13.49, *p* < 0.001, η*_p_^2^* = 0.11). There was no significant association between age and CBF (*r* = −0.15, *p* = 0.116; **Figure 1B**).

### Global Venous Oxygenation

There was no significant difference in global Y_v_ between BD (70.22 ± 5.74%) and HC adolescents (69.54 ± 5.75%) after controlling for age and sex (*F* = 0.89, *p* = 0.349, η*_p_^2^* = 0.008; **Figure 1C**). Age was a significant covariate (*F* = 4.90, *p* = 0.029, η*_p_^2^* = 0.042). Since there was no significant difference between groups, the association between Y_v_ and age was examined using the whole adolescent population (N = 115). Correlation analysis revealed that age had a significant effect on global Y_v_ (*r* = −0.19, *p* = 0.045; **Figure 1D**). Specifically, there was an age-related decline of global Y_v_ at a rate of 0.77% per year. Furthermore, there was a significant positive correlation between Y_v_ and CBF values across subjects (*r* = 0.42, *p* < 0.001).

### Global CMRO_2_

There was no significant difference in global CMRO_2_ between BD (156.27 ± 31.45 μmol/100 g/min) and HC adolescents (152.11 ± 33.74 μmol/100 g/min) when controlling for age and sex (*F* = 0.54, p = 0.47, η*_p_^2^* = 0.005; **Figure 1E**). Sex was a significant covariate (*F* = 20.67, *p* < 0.001, η*_p_^2^* = 0.157). Overall, female participants had higher CMRO_2_ compared to males (165.30 ± 33.0 vs. 139.79 ± 25.73 μmol/100 g/min, respectively; *t* = 4.51, *p* < 0.001, *d* = 0.86). There was no significant association between age and CMRO_2_ (*r* = 0.06, *p* = 0.50; **Figure 1F**).

### Relationship With Mood States

Global CBF, Y_v_, and CMRO_2_ were not significantly correlated with the total mood scores for mania (MRS) or depression (DEP-P) (N = 115, *p* > 0.05). To assess if the participants’ mood episode at the time of the MRI scan was associated with the cerebral metabolic measures, BD adolescents were further classified into three groups using their mood scores: presently hypomanic/mixed (MRS ≥12 with or without DRS ≥13; n = 23), presently depressed (DRS ≥13 and MRS ≤11; n = 17), and presently euthymic (DRS <12 and MRS ≤11; n = 18). Comparison of BD participants in the different mood states did not reveal any significant differences in the measurements for global Y_v_, CBF, and CMRO_2_ when controlling for age and sex (**Table 2**).

**Table 2:** CBF, Y_v_, and CMRO_2_ across mood states.^b^

Measure	BD hypomanic/ mixed (n = 23)	BD depressed (n = 17)	BD euthymic (n = 18)	Statistics		
				F	Partial *η*^2^	p
**CBF**	67.37 (12.36)	63.53 (13.78)	67.73 (10.46)	0.81	0.030	0.45
**Y** **_v_**	69.30 (5.20)	70.59 (6.97)	71.06 (5.26)	0.48	0.018	0.62
**CMRO** **_2_**	163.66 (27.48)	146.25 (35.61)	156.29 (31.17)	1.01	0.037	0.37

b CBF in ml/100g/min, Y_v_ in %O_2_, CMRO_2_ in μmol/100 g/min. Values are means ± SD. CBF, cerebral blood flow; Y_v_, venous blood oxygenation; CMRO_2_, cerebral metabolic rate of oxygen consumption.

Smoking is a potential confounding variable that might influence cerebral hemodynamics and oxygen metabolism. Sensitivity analysis was therefore conducted to examine the effect of excluding the smokers (n = 7) in the BD group. Exclusion of these participants did not change the results for any of the above analyses.

A large number of BD participants were using various medication including second-generation antipsychotics (SGAs; 60%) and lithium (22%; **Table 1**). To examine the effect of medication on the MRI measures, ANCOVA analysis was rerun covarying for medication usage in addition to age and sex. Current SGA use was a marginally significant covariate of Y_v_ (*F = 4.06, p = 0.046*) but not CBF or CMRO_2_. Lithium use was not significantly associated with any of the three measures.

## Discussion

To the best of our knowledge, this is the first study to characterize cerebral oxygen metabolism in BD as well as in an adolescent population. We hypothesized that individuals with BD would show alterations in baseline CMRO_2_ to reflect the change in CBF observed in prior studies (12, 39). Interestingly, our results demonstrate that although there is an increase in CBF among BD adolescents, the rate of global oxygen metabolism is unchanged. This imbalance between blood supply and oxygen consumption in the BD brain may reflect inefficiency in energy metabolism and the presence of some compensatory process to maintain CMRO_2_. Alternative interpretations of this finding are discussed herein.

We did not observe a group effect of CMRO_2_, which is to say that this largely global measure of oxygen consumption is not different between BD and HCs. The total GM CBF was significantly higher in BD compared to controls. This would in principle contribute to an increased CMRO_2_ in BD, but this CBF group effect was a fairly modest effect size, whereas Y_v_ was not significantly different between groups. Therefore, since CBF and Y_v_ are used together to calculate CMRO_2_, this helps to explain why there was no significant oxygen consumption group effect.

The relationship between CBF and CMRO_2_ has been the topic of investigation for several decades. It is widely accepted that there is tight coupling between CBF and CMRO_2_ at baseline (40). However, multiple studies have shown that there is an “uncoupling” between CBF and CMRO_2_ during neuronal activation (41–44). Specifically, the increase in CBF during activation has been observed to be significantly greater than the increase in CMRO_2_. In our study, although we were examining baseline physiology in the absence of neural stimulation, our results showed a significant increase in CBF without any changes in CMRO_2_, which appears to be similar to this phenomenon. If BD adolescents indeed have altered coupling between CBF and CMRO_2_ at baseline, it remains to be seen how this will affect CBF/CMRO_2_ coupling during neural stimulation. Our finding of an increase in CBF in the absence of alterations in CMRO_2_ can also be related to the phenomenon observed in hypercapnia experiments where there is a hypercapnia-induced increase in CBF with negligible change in CMRO_2_ (45, 46).

Another plausible interpretation of our results is that the increase in baseline CBF in BD might exist to serve functions other than oxygen metabolism. In addition to oxygen, the blood also carries and delivers glucose, the main source of energy for the brain. Similar to CMRO_2_, the cerebral glucose metabolic rate of glucose (CMR_glu_) represents another important index of neural function and energy homeostasis. However, unlike CMRO_2_, it is widely accepted that there is a tight coupling between CMR_glu_ and CBF during neural activation. Indeed, in their seminal work, Fox and colleagues demonstrated that the increase is CBF (50%) during neural activation is associated with a similar increase in CMR_glu_ (51%) but a far more muted increase in CMRO_2_ (5%) (42). Thus, it is possible that the increase in CBF observed in our BD participants might be related to alterations in CMR_glu_.

Molecular studies examining mitochondrial function and oxidative stress provide strong evidence showing that energy metabolism is disturbed in BD (18, 47). In addition, studies have reported elevated lactate concentration in the brain (19) and cerebrospinal fluid (20) of individuals with BD. Furthermore, metabolomic analysis has found increased serum levels of pyruvate, the end product of glycolysis and the main fuel input for the citric acid cycle (CAC), in BD patients (48). Taken together, these findings suggest that there is a shift in metabolism from oxidative phosphorylation to the less-efficient glycolysis pathway in the brain of BD patients (49). Whereas the complete oxidation of glucose yields large amounts of energy in the form of ATP (30–36 ATP), glycolysis only produces 2 ATP (50). Thus, the brain of BD individuals may require more fuel in the form of glucose to meet the high energy demands of the brain. The increase in CBF observed in our study might reflect a compensatory mechanism in place to increase glucose delivery to the brain of BD participants. This is in line with our hypothesis discussed earlier that the increase in CBF observed in BD participants may serve functions other than oxygen metabolism. Future studies that relate CMRO_2_ with arterial lactate-to-glucose ratios in BD are warranted.

Although we did not observe a difference in global CMRO_2_ between groups, it is possible that alterations in oxygen metabolism may exist and are localized to specific brain regions in BD. Multiple studies have demonstrated structural changes, more specifically GM volume reduction in specific regions of the brain (i.e. anterior cingulate cortex, middle frontal gyrus) of patients with mood disorders (51, 52). Furthermore, in our prior study examining CBF in BD adolescents, we found that CBF was significantly increased and localized in brain regions including the medial frontal and middle cingulate regions compared to HCs (12). Thus, it is can be expected that these brain regions that show structural changes and region-specific elevation in CBF will also have localized changes in energy metabolism. The current TRUST MRI acquisition provides only a global estimate of CMRO_2_; thus, we are unable to provide insight on regional changes.

A recent study from our group identified that CBF is altered according to mood states in adolescents with BD (39). Therefore, in the present study, we similarly divided our BD participants into three groups, euthymic, depressed, or manic/hypomanic/mixed, to explore the possibility that CMRO_2_ might also vary by mood state. We did not detect any significant differences in CBF, Y_v_, or CMRO_2_ between the three BD groups. As discussed earlier, it is likely that alterations in these physiological parameters may be localized rather than occur at a global level. Indeed, the mood state–related changes in CBF observed in the study mentioned were localized to certain brain regions (i.e. anterior cingulate cortex).

We observed an age-related decline in Y_v_, which is a novel finding for this age group but is consistent with results from previous studies on normal adult aging (21, 28). This indicates that a greater fraction of oxygen is extracted by the brains of older individuals, which suggests alterations in oxygen demand with age. Interestingly, the rate of decline observed in our adolescent population is much greater (0.77% per year) than that reported in one study conducted on an older population (0.14% per year in ages 20–89 years) (21). This may be reflected by the fact that the developing brain undergoes extensive metabolic changes that persist until 16–18 years of age (53). In contrast to Y_v_, we did not observe any significant association between age and CBF or age and CMRO_2_. A number of studies have previously reported a decline in CBF with age (22, 54, 55). While our age range spanned early adolescence to young adulthood (14–21 years), in actuality, the vast majority of participants were in mid-adolescence (17.3 ± 1.4 years); as such, the age variance across participants was likely not sufficient to detect an effect of age on CBF. We observed a significant positive association between CBF and Y_v_, which is consistent with prior findings in adults (56). Literature on age-related changes in CMRO_2_ is inconsistent, with some studies reporting increased CMRO_2_ with age (21, 22), while others have reported no change (57, 58) or even a decrease in CMRO_2_ across the life span (37, 59).

We found that adolescent females have greater CBF and CMRO_2_ compared to males. This finding is in line with recent studies conducted on older healthy cohorts (21, 22). Although it is not fully understood why such sex differences exist, a few hypotheses have been proposed. First, as females tend to have lower hematocrit values, it is thought that more CBF might be needed to carry an equivalent amount of oxygen as compared to males (21, 60). Others have attributed the higher baseline metabolic rate in women to the effect of hormones (i.e. estrogen) (61). In addition, differences in brain structure and size might also be a factor that reflects the observed sex-related differences.

There are some limitations to address in the present study. As described earlier, the TRUST MRI technique is limited to global estimations of Y_v_ and CMRO_2_, as measurement is confined to a large terminal draining veins (i.e. SSS). Thus, we were unable to obtain information on any potential regional metabolic changes. Moreover, it is important to note that although our ROI for TRUST, the SSS, drains most of the cerebral cortex, not all of the cerebral blood is drained through this sinus (e.g. deep cerebral regions drain through the straight sinus instead). Therefore, Y_v_ might be underestimated in deeper cerebral regions. Second, our estimate of global CBF was based solely on ROI analysis of GM regions and does not account for CBF in white matter. Third, we did not measure Y_a_ using pulse oximetry for participants in this study and therefore estimated it to be a constant value of 99% based on physiological literature. The effect of this is likely to be minimal, as Y_a_ has been observed to be close to unity and minimally affected by age (21, 37). Similarly, we estimated hematocrit values (used for T2 calibration for TRUST) using laboratory reference values accounting for both age and sex instead of blood sampling to keep our study procedures completely noninvasive. Lastly, a large portion of our BD participants were using medication that might have impacted our findings. Indeed, medication use has previously been shown to influence CBF as well as cerebral metabolism (62–64).

## Conclusion

In conclusion, this study demonstrates that cerebral oxygen utilization is not altered in adolescents with BD, despite the increased CBF observed in this population. Our results provide new insight on baseline energy homeostasis processes in BD. Furthermore, we found sex differences in that female adolescents overall have higher CBF and CMRO_2_ than males. In addition, a rapid decrease of Y_v_ from adolescence to young adulthood was observed. Although the TRUST technique used herein offers several advantages that include noninvasiveness, speed (∼1.5 min scan), and feasibility in children and adolescents, it is limited to global estimations of Y_v_ and CMRO_2_. Research is currently underway to develop noninvasive MRI techniques for 3-D brain oxygenation mapping that permits Y_v_ quantification in both large sinuses as well as smaller cortical veins (65). Future studies that use these novel techniques are warranted to examine if there are any region-specific metabolic changes in BD.

## Data Availability Statement

The datasets generated for this study will not be made publicly available. We did not obtain consent from our participants for sharing of data when the study was conceived, so we cannot provide raw participant-level data.

## Ethics Statement

The studies involving human participants were reviewed and approved by Sunnybrook Health Sciences Centre Research Ethics Board. Written informed consent to participate in this study was provided by the participants’ legal guardian/next of kin.

## Author Contributions

SK performed data processing and analysis, interpreted results, and prepared the figures and manuscript. LF assisted with statistical analysis and revised the manuscript. AG helped with data processing and revised the manuscript. HL provided technical guidance and revised the manuscript. BG and BM contributed to study conception and experimental design and assisted with manuscript preparation.

## Funding

This research was supported by a grant to BIG from the Ontario Mental Health Foundation and Canadian Institutes of Health Research (CIHR). BJM received a National Alliance for Research on Schizophrenia & Depression (NARSAD) Independent Investigator Award from the Brain and Behavior Foundation.

## Conflict of Interest

The authors declare that the research was conducted in the absence of any commercial or financial relationships that could be construed as a potential conflict of interest.
